# Leaf functional traits and resource use strategies facilitate the spread of invasive plant *Parthenium hysterophorus* across an elevational gradient in western Himalayas

**DOI:** 10.1186/s12870-024-04904-0

**Published:** 2024-04-02

**Authors:** Padma Sharma, Sonia Rathee, Mustaqeem Ahmad, Manzer H. Siddiqui, Saud Alamri, Shalinder Kaur, Ravinder K. Kohli, Harminder Pal Singh, Daizy R. Batish

**Affiliations:** 1 Department of Environment Studies, Panjab University, Chandigarh, 160014 India; 2 Department of Botany, Panjab University, Chandigarh, 160014 India; 3 Department of Botany and Microbiology, College of Science, King Saud University, Riyadh, 11451 Saudi Arabia; 4 Amity University, Sector 82A, IT City, International Airport Road, Mohali, 140 306 India

**Keywords:** Leaf functional traits, Nutrient content, Specific leaf area, Trait‒trait relationship

## Abstract

*Parthenium hysterophorus* L. (Asteraceae) is a highly prevalent invasive species in subtropical regions across the world. It has recently been seen to shift from low (subtropical) to high (sub-temperate) elevations. Nevertheless, there is a dearth of research investigating the adaptive responses and the significance of leaf functional traits in promoting the expansion to high elevations. The current study investigated the variations and trade-offs among 14 leaf traits (structural, photosynthetic, and nutrient content) of *P. hysterophorus* across different elevations in the western Himalayas, India. Plots measuring 20 × 40 m were established at different elevations (700 m, 1100 m, 1400 m, and 1800 m) to collect leaf trait data for *P. hysterophorus*. Along the elevational gradient, significant variations were noticed in leaf morphological parameters, leaf nutrient content, and leaf photosynthetic parameters. Significant increases were observed in the specific leaf area, leaf thickness, and chlorophyll *a*, total chlorophyll and carotenoid content, as well as leaf nitrogen and phosphorus content with elevation. On the other hand, there were reductions in the amount of chlorophyll *b*, photosynthetic efficiency, leaf dry matter content, leaf mass per area, and leaf water content. The trait-trait relationships between leaf water content and dry weight and between leaf area and dry weight were stronger at higher elevations. The results show that leaf trait variability and trait-trait correlations are very important for sustaining plant fitness and growth rates in low-temperature, high-irradiance, resource-limited environments at relatively high elevations. To summarise, the findings suggest that *P. hysterophorus* can expand its range to higher elevations by broadening its functional niche through changes in leaf traits and resource utilisation strategies.

**Supplementary Information:**

The online version contains supplementary material available at 10.1186/s12870-024-04904-0.

## Background

 The response of an invasive species to climate change is contingent upon its functional ecology [1]. Functional trait responses in plants serve as valuable indications of their successful adaptation to both biotic and abiotic elements of their environment [2–4]. Plant functional traits play a significant role in controlling growth rate, mortality, and dispersal over different time periods and regions [5]. It is, therefore, crucial to comprehend how invasive plants adapt to environmental gradients by altering their functional traits [6]. Leaves, which are directly involved in respiration, transpiration, and carbon fixation, play a vital role in ensuring the long-term survival of plants [7]. Modifications in the functional attributes of leaves, including leaf shape, lamina area, biomass, water content, and specific leaf area (SLA), significantly impact growth and development, particularly in response to fluctuations in environmental resources [4, 8]. The nutrient content in the leaves may provide insight into the nutrient utilisation strategy of a particular species [9, 10]. Leaf traits and their trade-offs can be linked to invasion success along an elevational gradient since they play an important role in influencing plant performance and fitness in diverse environments [11, 12].

In particular, leaf area, SLA, and leaf thickness can influence plant growth, carbon balance, and water use efficiency and are critical factors in determining the ability of a plant to establish and sustain itself in a new environment [12]. A small leaf area restricts the absorption of incident solar radiation at higher elevations, and the rate of evapotranspiration lessens the harm from UV radiation and strong winds [7, 13]. SLA is an important trait that influences plant tolerance, competitiveness [8], leaf lifespan, photosynthetic capacity, and growth rate [14, 15]. In resource-constrained environments, such as those found at higher elevations, invasive plants may have a competitive advantage due to leaf traits associated with high SLA and high photosynthetic rates [4]. The correlation between leaf traits and invasion success along an elevational gradient can be complex, contingent upon the environmental conditions and biotic interactions that exist at each site. Thus, to ascertain how an invasive species adjusts to elevation, an in-depth investigation of the functional attributes of its leaves is required [16].

*Parthenium hysterophorus* L. (ragweed parthenium; Asteraceae) is an invasive herbaceous plant native to Mexico, the Caribbean, Central America, and South America [17, 18]. The species is presently distributed throughout tropical, subtropical, and semiarid regions and is also found in warm and sub-temperate areas [19, 20]. Strong colonisation abilities, a robust seed bank, high propagule pressure, resistance to a broad spectrum of temperature and water stresses, and an annual life cycle all contribute to the high invasion potential of *P. hysterophorus* [19, 21, 22]. In Nepal, *P. hysterophorus* has been found growing at heights of up to 2,000 m above sea level [23], and it has been proposed that climate change may significantly increase this elevation. Rathee et al. [20] found that *P. hysterophorus* migrates to higher elevations due to an increase in biomass allocated to reproductive organs. The ability of the plant to modify its aboveground height, root biomass, capitula count, and seed mass also has an impact on this migration. However, variations in leaf traits and associated trade-offs might affect the ability of *P. hysterophorus* to adapt to hilly environments. Investigations into the trait-trait scaling interactions of invading species are necessary to comprehend the mechanisms underpinning adaptability in invaded ecosystems [24]. On the basis of this rationale, we hypothesized that examining qualitative traits might provide insights into the role of leaf morphological and ecophysiological characteristics in facilitating invasion at higher altitudes. Therefore, the objective of this study was to analyse the variability and interdependence (trade-offs) of fourteen functional leaf traits (structural, photosynthetic, and nutritional attributes) of *P. hysterophorus* at altitudes ranging from 700 to 1,800 m in the western Himalayas, India. The traits studied in this research were: leaf area, leaf dry matter content, specific leaf area, leaf mass per area, leaf thickness, leaf dry weight (structural attributes), total chlorophyll content, chlorophyll *a* and *b* and total carotenoid content, photosynthetic efficiency (photosynthetic capacity), total nitrogen and total phosphorus content, and leaf water content (nutrient content). Studying various plant traits, especially leaf attributes, aids in a better understanding of the invasion process [25]. The study will contribute to the understanding of how invasive plants adapt by altering their ecophysiological traits and offer a theoretical and functional approach for forecasting non-native plants with the highest likelihood of becoming invasive.

## Methods

### Study system

**Fig. 1:**
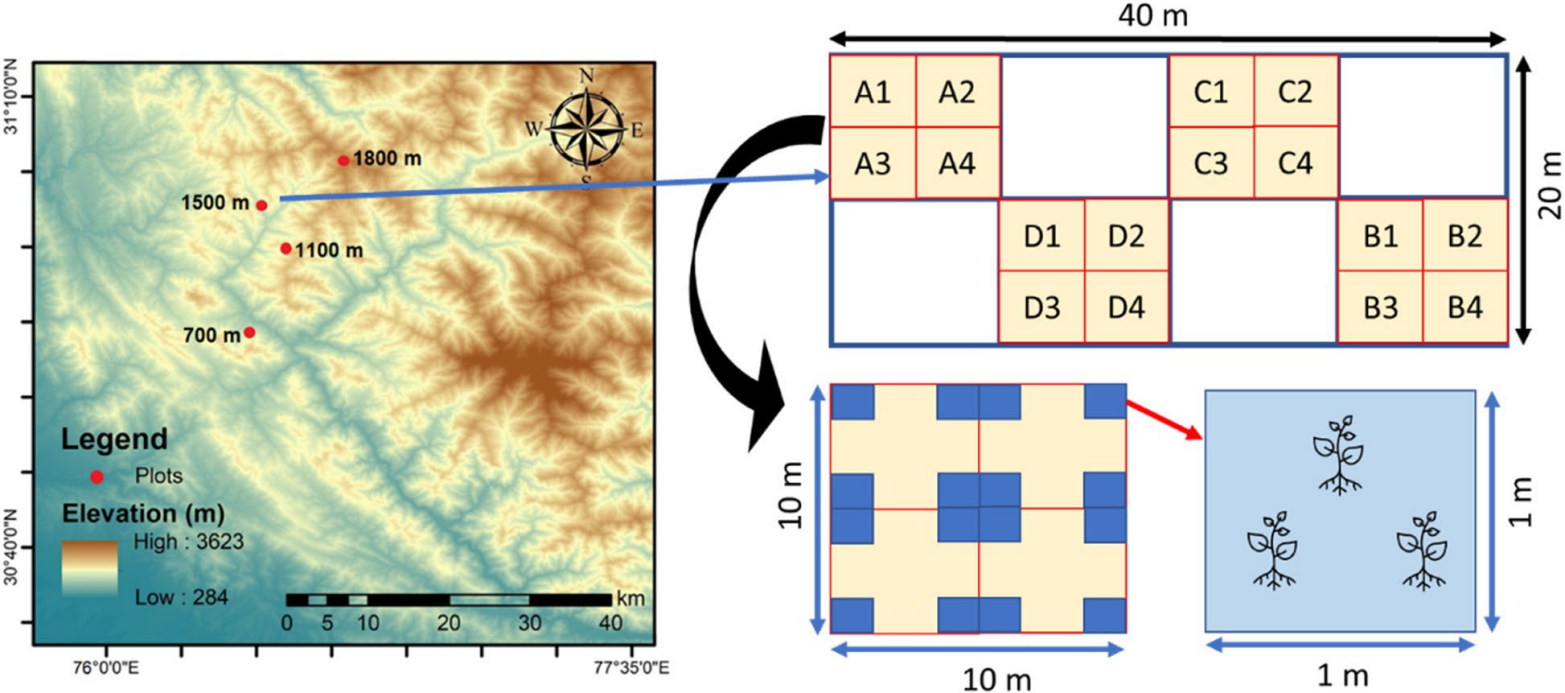
Map of the study area representing study sites at different elevations (700 m, 1100 m, 1500 m, and 1800 m) in the western Himalayas, India, and sketch diagram showing plot establishment and plant selection method for leaf trait measurement in the study sites

 The study was carried out at an elevation gradient of 700 to 1,800 m above sea level in the western Himalayan Shiwalik ranges (Fig. 1) in the years 2021–2022. The study sites were located along a transect between 30° 50′ 101′′N, 76° 57′ 913′′E (700 m) and 31° 02′ 053′′N, 77° 079′ 198′′E (1,800 m) in two districts (Solan and Shimla) of Himachal Pradesh (India). In the Solan district, the temperature fluctuated between 0.6 and 32.2 °C, with an average annual precipitation of 1140.86 mm. In the Shimla district, the temperature varied from 2.5 to 26°C, with an average annual precipitation of 999.64 mm (source: https://www.cgwb.gov.in/sites/default/files/2022-10/shimla.pdf). Human habitation and agricultural practices are the primary causes of disturbance at lower elevations (up to 900 m), and a sizable portion of the local flora is made up of non-native species [26]. However, at higher elevations (> 1000 m), where agriculture, the lumber industry, grazing land, and tourism are vital, subtropical pine with broadleaf forest is more prevalent [26].

### Site selection and plot layout

Study sites were chosen at regular intervals of 300–400 m in the selected transect. Plots measuring 20 × 40 m (Fig. 1) were established at elevations of 700 m, 1,100 m, 1,400 m, and 1,800 m, with a distance of 50 m from the roadside. Roadside locations can serve as routes for the introduction and dissemination of invasive species. Human activities, such as transportation and construction, can contribute to the dispersal of invasive plants. Studying plots located along roadsides helps evaluate the possible influence of these pathways on the spread of non-native species [27]. These plots were split into 10 × 10 m for the documentation of shrubs and 1 × 1 m for recording herbaceous species. The vegetation composition of each plot was examined using a systematic quadrat sampling approach. An analysis of the vegetation composition revealed that, except for the plot at 1,800 m, *P. hysterophorus* is the most common invasive species, covering ≥ 20% of the elevational gradient. Professor R.K. Kohli validated the plant, and a voucher specimen (Voucher # PAN 21463) was deposited in the herbarium at Panjab University in India. The arrangement of plots and plant selection from each 1 × 1 m quadrat are described in Fig. 1. The plant material was collected from the selected sites. The measurements were taken when the plants were at a robust developmental stage. Since the collected plants were growing wild in the selected sites or plots and these were not on any private or government recognized forest land, no permission is required. Each site included a mixture of shrubs (*Lantana camara* L., *Xanthium strumarium* L., and *Calotropis procera* (Aiton) Dryand.) and herbs (*Solanum nigrum* L., *Plantago major* L., *Solanum tuberosum* L., *Senna tora* (L.) Roxb., *Sonchus asper* (L.) Hill, *Abutilon indicum* (L.) Sweet, *Medicago sativa* L., *Cannabis sativa* L., *Achyranthes aspera* L., *Oxalis corniculata* L., and *Capsella bursa-pastoris* (L.) Medik) with scattered trees (*Acacia nilotica* (L.) Delile, *Albizia julibrissin* Durazz., *Pinus* spp., *Leucaena leucocephala* (Lam.) de Wit, and *Prosopis juliflora* (Sw.) DC.).

### Quantification of leaf traits and nutrient content

In the current investigation, adult *P. hysterophorus* plants that showed consistent development and were not obstructed by other plants were selected for the assessment of leaf traits. To evaluate functional trait values, we selected three healthy, mature plants from each 1 × 1 m quadrat, tagged one leaf per plant, and computed an average of those values for each quadrat. In other words, one leaf trait value was acquired for each 1 × 1 m quadrat containing *P. hysterophorus*. The leaves that looked healthy, had intact lamina, were exposed to the sunlight, and exhibited no symptoms of disease or pest infestation were chosen and tagged. The leaf traits examined in the study were leaf area (LA; mm^2^), leaf dry matter content (LDMC; mg g^−1^), specific leaf area (SLA; mm^2^ mg^−1^), leaf mass per area (LMA; mg mm^−2^), leaf thickness (LT; mm), and leaf dry weight (LDW; mg). The lamina of the tagged leaves was measured in the field using a leaf area metre (CI-202; CID Bio-Science, USA). The tagged leaves were collected, weighed, and labelled based on the elevation at each location, and transported to the lab to determine the leaf water content (LWC; mg) and LDW. The leaves were dried for 72 h at 60 °C in a hot air oven before being weighed with an electronic weighing balance (A&D Co., Japan; accuracy = 0.10 mg). The leaf water content was measured by calculating the difference between the weight of the fresh leaf and the weight of the dry leaf. Leaf mass per area (LMA) was calculated as the leaf dry mass per unit leaf area, and the SLA was calculated by dividing the leaf area by its dry mass [28]. The formulas used for calculating various quantitative leaf traits are given in Table S1, Supplementary material. Leaf thickness was measured using a digital Vernier calliper with an accuracy of 0.01 mm. For every elevational site, leaf samples were collected, dried, and homogenised. The acid digestion method was used to assess the total nitrogen (mg g^−1^) and total phosphorus (mg g^−1^) contents of the leaf samples [29].

### Assessment of photosynthetic features

The amounts of total chlorophyll (TChl; µg mg^−1^), chlorophyll *a* and *b* (Chl *a* and Chl *b*; µg mg^−1^), total carotenoid (TCaro) content (µg mg^−1^), and photosynthetic efficiency (Fv/Fm) were quantified in the selected plant leaves. The photosynthetic efficiency of the labelled leaves was quantified in the field using a pulse-modulated chlorophyll fluorometer (OS-30p; OptiSci., USA). Chlorophyll was extracted from 20 mg of fresh leaves in 4 mL of dimethyl sulfoxide. The resulting solution was subsequently incubated at 60 °C for one hour, following the methodology described by Hiscox and Israelstam [30]. The absorbance of the extractant was measured at 645, 663, and 470 nm using a Shimadzu UV-1800 spectrophotometer, with dimethyl sulphoxide as a blank. TChl, Chl *a*, and Chl *b* contents were determined according to the formulas given by Arnon [31]. In addition, the TCaro content was determined using the methodology outlined by Lichtenthaler and Wellburn [32].

### Statistical analyses

The impact of elevation on the leaf traits of *P. hysterophorus* was investigated employing linear regression models. When linear regression models were deemed inadequate for fitting the data, quadratic regression models were applied to optimise model performance. In all the models, elevation functioned as a numerical predictor, while the trait characteristics of each group at different elevations were considered the dependent variables. After verifying the normal distribution of the data, regression models were applied. The standardised major axis (SMA) regressions were used to examine the trade-offs among the functional traits of leaves, as it is a widely employed statistical tool commonly used for allometric investigations [33]. The ‘*smatr*’ software was used to estimate SMA regressions using trait data from each elevational population. SMA was done to determine the best-fitting scaling relationship between two traits on a log-log axis. In addition, the coefficient of determination (R^2^) for SMA was plotted across the range of elevations using either linear or polynomial regression. The statistical analyses were carried out using R software (version 4.1.2) developed by the R Core Team.

## Results

### Variation in leaf functional groups along an elevation gradient

The multivariate analysis of variance, using Pillai’s test, showed substantial differences in leaf morphological traits and nutrient content (F_(1,3)_ = 27.9, *p* < 0.001), as well as leaf photosynthetic parameters (F_(1,3)_ = 14.16, *p* < 0.001), along the elevational gradient.

### Leaf morphological features vary across different elevations

**Fig. 2:**
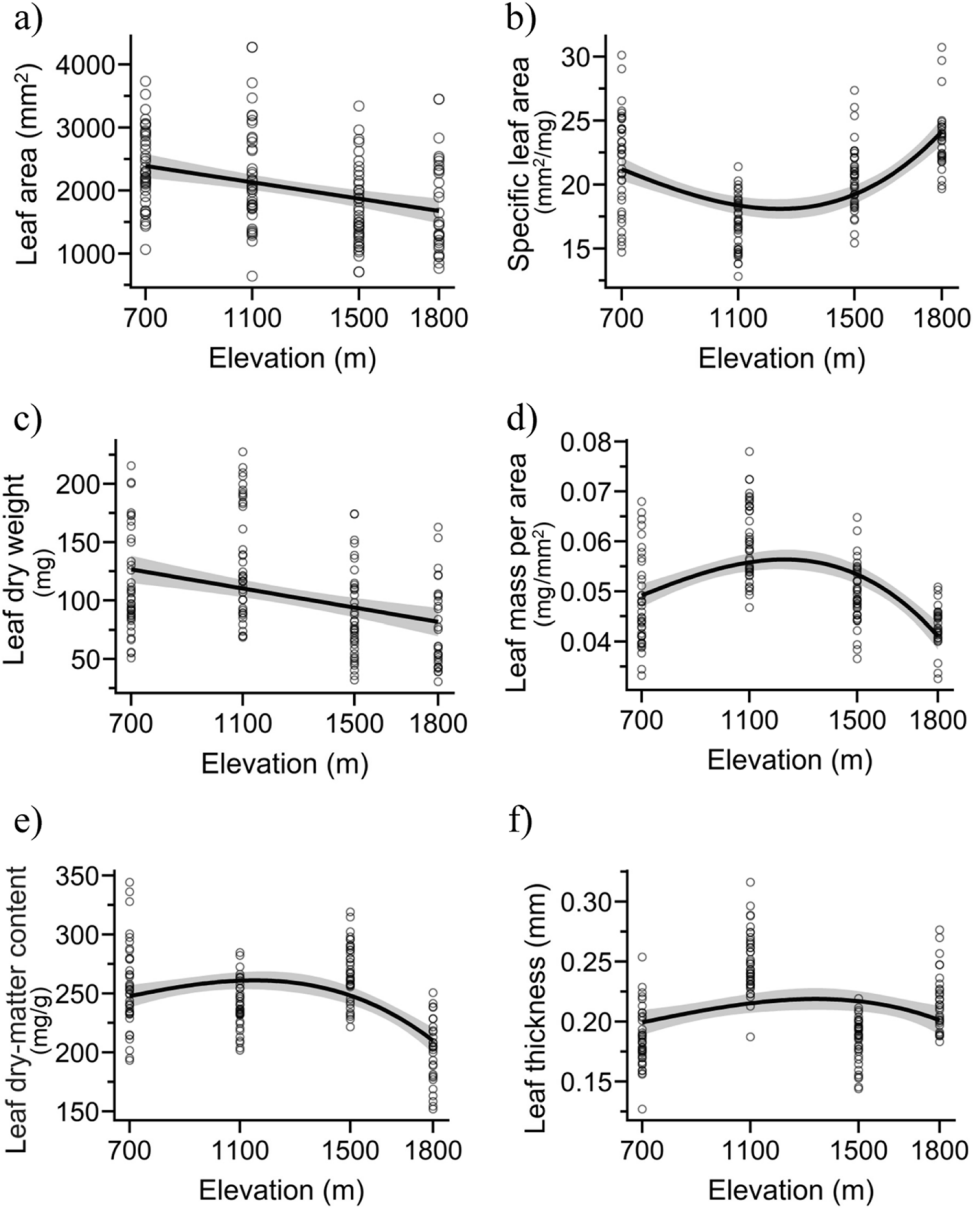
Pattern of leaf functional traits of *Parthenium hysterophorus* across an elevation gradient: **a** Leaf area; **b** Specific leaf area; **c** Leaf dry weight; **d** Leaf mass per area; **e** Leaf dry-matter content; and **f** Leaf thickness. The 95% confidence interval is represented by the shaded area, and the model patterns are represented by the line

All the leaf morphological traits that were investigated exhibited significant variations along the elevational gradient (Table 1). With elevation, SLA showed an inverse hump-shaped pattern (Fig. 2b). LA and LDW declined linearly with increasing elevation (Fig. 2a, c), while LMA, LDMC, and leaf thickness exhibited a hump-shaped pattern with increasing elevation (Fig. 2d, e, f). The model-adjusted *R*^2^ values were highest for SLA and LMA (*R*^2^ = 0.36, *p* < 0.001) (Table 1). The leaf area (LA) was measured to be greatest at an altitude of 700 m (2,357.5 ± 97.8 mm^2^), while it was 1,727.3 ± 100.1 mm^2^ at 1,500 m (Fig. 1a). LDW at an altitude of 1,100 m was measured to be 133.8 ± 7.53 mg, whereas at an altitude of 1,800 m, it was found to be 76.5 ± 6.58 mg (Fig. 1b). The minimum specific leaf area (SLA) was measured at an elevation of 1,100 m (16.98 ± 0.33 mm^2^ mg^−1^), while the maximum SLA was observed at 1,800 m (23.52 ± 0.46 mm^2^ mg^−1^). At an elevation of 1,100 m, the LMA was the highest (0.06 ± 0.001 mg mm^−^^2^), whereas at 1,800 m, it was the lowest (0.043 ± 0.001 mg mm^−^^2^). The LDMC at an altitude of 1,500 m was approximately 267.5 ± 3.80 mg g^−1^, whereas at an altitude of 1,800 m it was approximately 200.7 ± 4.89 mg g^−1^.

**Table 1:** Linear regression models showing functional trait variations in *Parthenium hysterophorus* across an elevational gradient

Response variables	Predictor	Estimate	Std. Error	t-value	* p*-value	Multiple *R*^2^	Adjusted *R*^2^
Leaf area (mm^2^)	(Intercept)	2843.62	187.68	15.15	< 0.001	0.12	0.12
	Elevation	−0.65	0.14	−4.51	< 0.001		
Leaf dry weight (mg)	(Intercept)	155.29	11.5	13.49	< 0.001	0.13	0.12
	Elevation	−0.04	0.01	−4.64	< 0.001		
Specific leaf area (mm^2^ mg^−1^)	(Intercept)	40.3	2.6	15.50	< 0.001	0.37	0.36
	Elevation	0.0	0.0	8.85	< 0.001		
Leaf mass per area (mg mm^−2^)	(Intercept)	0.0	0.01	0.28	0.78	0.37	0.36
	Elevation	0.0	0.0	−8.66	< 0.001		
Leaf dry-matter content (mg g^−1^)	(Intercept)	165.0	28.8	5.72	< 0.001	0.20	0.19
	Elevation	0.0	0.0	−4.19	< 0.001		
Leaf thickness (mm)	(Intercept)	0.08	0.03	2.69	< 0.001	0.11	0.10
	Elevation	0.0	0.0	−4.19	< 0.001		
Photosynthetic efficiency (*F*_*v*_*/F*_*m*_)	(Intercept)	0.71	0.02	45.13	< 0.001	0.09	0.09
	Elevation	−0.0	0.0	−3.86	< 0.001		
Chlorophyll *a* (µg mg^−1^)	(Intercept)	−4.97	1.89	−2.64	< 0.01	0.44	0.43
	Elevation	0.0	0.0	−5.76	< 0.001		
Chlorophyll *b* (µg mg^−1^)	(Intercept)	0.7	0.09	8.38	< 0.001	0.06	0.06
	Elevation	0.0	0.0	−3.15	< 0.001		
Total chlorophyll content (µg mg^−1^)	(Intercept)	−4.76	2.17	−2.19	0.03	0.40	0.39
	Elevation	0.0	0.0	−5.52	< 0.001		
Total carotenoid content (µg mg^−1^)	(Intercept)	−1.37	0.5	−2.76	< 0.01	0.48	0.48
	Elevation	0.0	0.0	−8.15	< 0.001		
Leaf water content (mg)	(Intercept)	0.4	0.04	10.27	< 0.002	0.04	0.03
	Elevation	0.0	0.0	−2.40	0.02		
Leaf nitrogen content (mg g^−1^)	(Intercept)	3.62	0.03	113.27	< 0.001	0.81	0.81
	Elevation	0.0	0.0	−25.25	< 0.001		
Leaf phosphorus content (mg g^−1^)	(Intercept)	44.56	0.47	95.47	< 0.001	0.87	0.87
	Elevation	−0.01	0.0	−31.4	< 0.001		

Data is reported up to only two decimal places

The LDMC declined with elevation, except at 1,500 m. It was highest (255.9 ± 5.61 mg g^−1^) at 700 m elevation and the lowest (200.7 ± 4.89 mg g^−1^) at 1,800 m. A marginal increase in leaf thickness was observed along the elevation gradient.

### Elevation-dependent variations in leaf photosynthetic properties

**Fig. 3:**
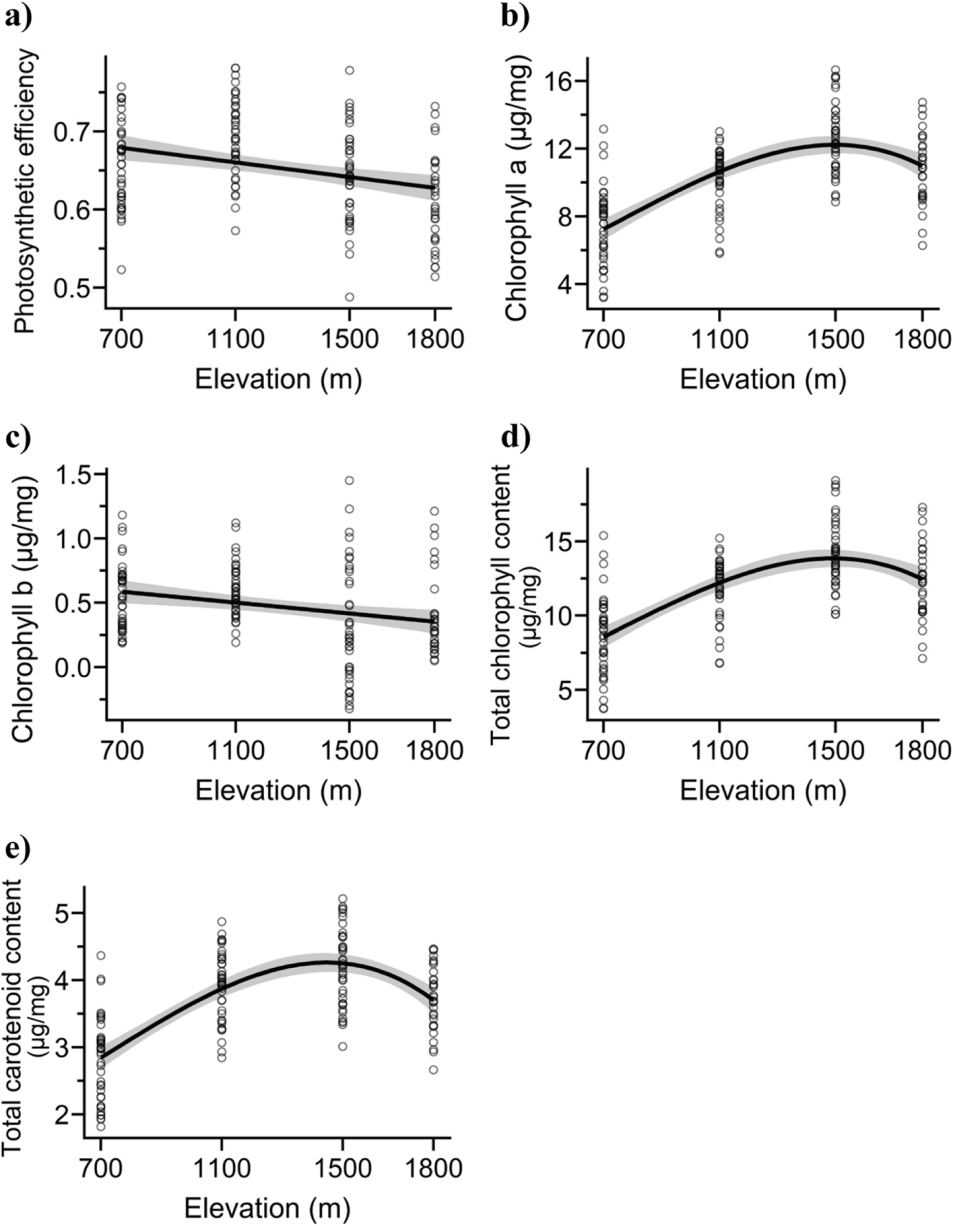
Pattern of *Parthenium hysterophorus* leaf photosynthetic characteristics: **a** photosynthetic efficiency, **b** chlorophyll *a*, **c** chlorophyll *b*, **d** total chlorophyll content, and **e** total carotenoid content across an elevational gradient. The 95% confidence interval is represented by the shaded area, and the model patterns are represented by the line

Chlorophyll *b* and photosynthetic efficiency decreased linearly with elevation, but the contents of Chl a, TChl, and TCaro showed a hump-shaped pattern with elevation (Fig. 3). Total carotenoid had the highest model-adjusted *R*^2^ value (*R*^2^ = 0.48, *p* < 0.001), followed by Chl *a* (*R*^2^ = 0.43, *p* < 0.001) and total chlorophyll (*R*^2^ = 0.39, *p* < 0.001), while Chl *b* had the lowest value (*R*^2^ = 0.06, *p* < 0.001) (Table 1). Along the 700–1,800 m range, the photosynthetic efficiency was between 0.61 and 0.69, the Chl *a* concentration was between 7.40 and 12.61 µg mg^−1^, and the Chl *b* concentration was between 0.32 and 0.60 µg mg^−1^. The TChl ranged from 8.71 to 14.19 µg mg^−1^, and TCaro ranged from 2.82 to 4.20 µg mg^−1^.

### Leaf water and nutrient levels vary at different elevations

**Fig. 4:**
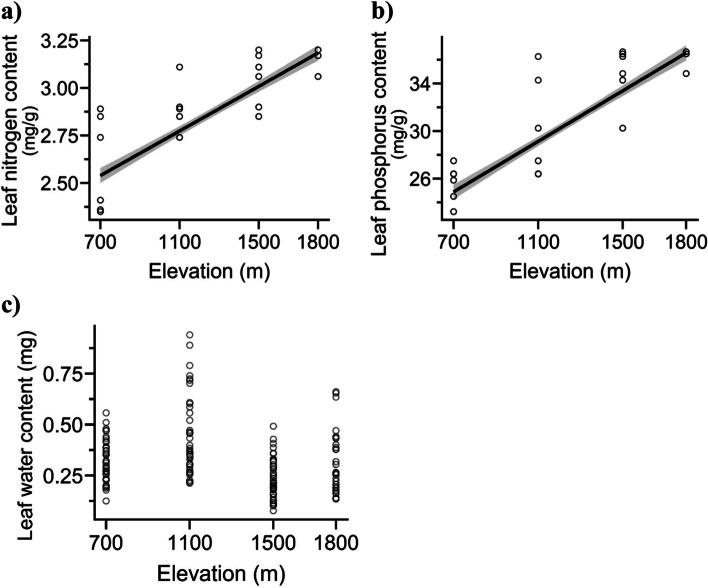
Pattern of (**a**) leaf nitrogen content, **b** leaf phosphorus content, and **c** leaf water content of *Parthenium hysterophorus* across an elevational gradient. The 95% confidence interval is represented by the shaded area, and the model patterns are represented by the line

As the elevation increased, there were significant changes in the leaf water content, nitrogen content, and phosphorus content (Table 1). The leaf nutrient content had a positive correlation with elevation, although leaf water content demonstrated a negative correlation (Fig. 4). The highest leaf water content (0.44 ± 0.30 mg) was recorded at an altitude of 1,100 m, whereas the lowest (0.24 ± 0.02) was found at 1,500 m. The highest leaf nitrogen content (3.15 ± 0.06 mg g^−1^) was measured at an altitude of 1800 m, while the lowest (2.37 ± 0.03 mg g^−1^) was observed at 700 m. Similarly, the highest phosphorus content (36.01 ± 0.83 mg g^−1^) was found at 1800 m, whereas the lowest (25.00 ± 1.09 mg g^−1^) was recorded at 700 m (Fig. 4c).

### Comparing the exponents of scaling in leaf functional features

**Fig. 5:**
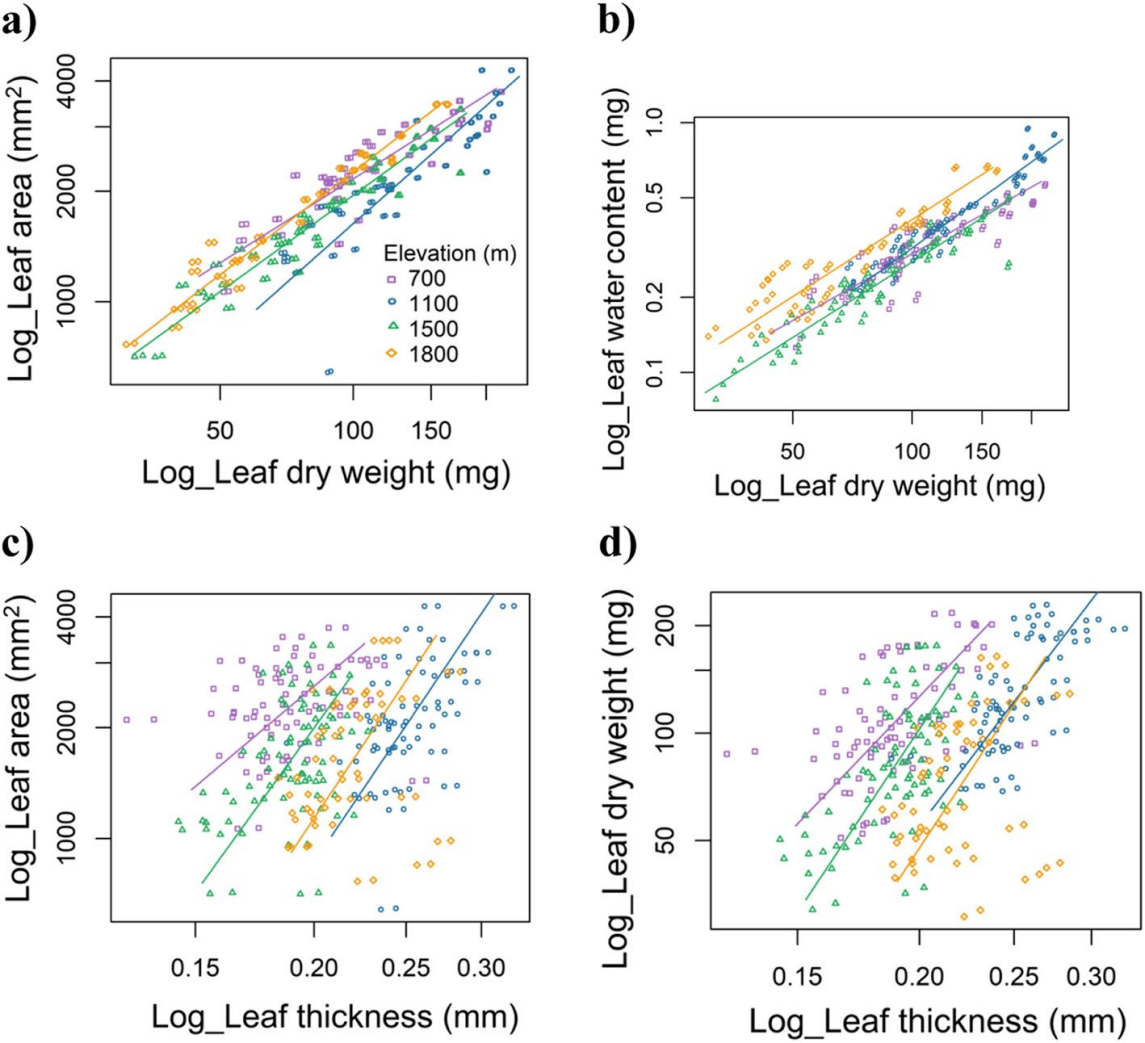
Standardized major axis (SMA) regressions depicting the relationships between leaf functional traits across an elevational gradient. **a** leaf area (Log_LA) ~ leaf dry weight (Log_LDW), **b** leaf water content (Log_LWC) ~ leaf dry weight (Log_LDW), **c** leaf area (Log_LA) ~ leaf thickness (Log_LT), and **d** leaf dry weight (Log_LDW) ~ leaf thickness (Log_LT)

**Fig. 6:**
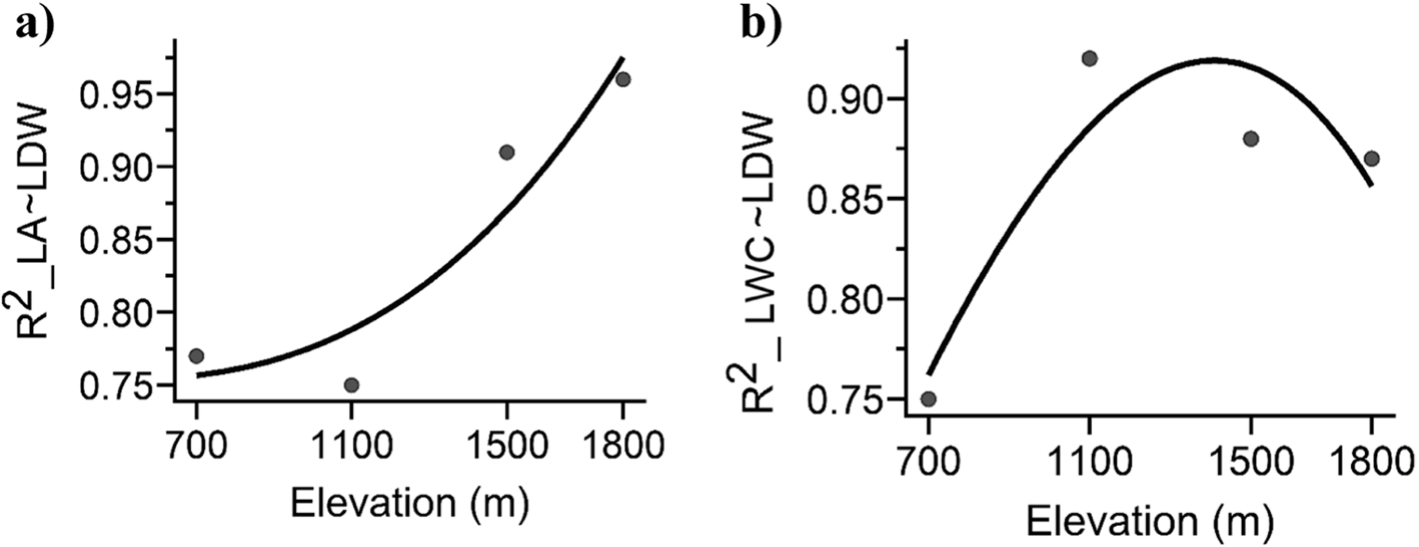
Pattern of *R*^2^ values of the scaling exponents of **a** LA ~ LDW and **b** LWC ~ LDW with elevation. LA = leaf area; LDW = leaf dry weight; and LWC = leaf water content

 At each elevation, the four bivariate relationships between LA and LDW, LWC and LDW, LA and LT, and LDW and LT exhibited variations in both steepness and size (Fig. 5; Table 2). The bivariate linkages at each elevation exhibited positive relationships, as depicted in Fig. 5a–d. The coefficient of determination (R^2^) for the association between LA and LDW was significantly higher at an elevation of 1,800 m (*R*^2^ = 0.96, *p* < 0.001) than it was at a lower elevation of 1,100 m (*R*^2^ = 0.75, *p* < 0.001) (Table 2). The value of *R*^2^ for LWC ~ LDW was highest at an altitude of 1,100 m (*R*^2^ = 0.92, *p* < 0.001) and lowest at 700 m (R^2^ = 0.75, *p* < 0.001). The highest *R*^2^ value for the LA ~ LT relationship was observed at an altitude of 1,100 m (*R*^2^ = 0.27, *p* < 0.001), while the lowest value (*R*^2^ = 0.04, *p* < 0.001) was recorded at 700 m. The LDW ~ LT was found to have a maximum value at 1,100 m (*R*^2^ = 0.49, *p* < 0.001) and a minimum value at 1,800 m (*R*^2^ = 0.08, *p* < 0.001) (Table 2). In addition, the relationship between LA and LDW was not significant at 700 m and 1,800 m (Fig. 6a, b). At 1,800 m, the association between LDW and LT was equally non-significant. Except at 1,100 m elevation, LA increased gradually and disproportionately as LDW (slope [α] < 1) increased (Table 2). Except for LWC ~ LDW at 700 m, slope values (α) > 1 were recorded for LWC ~ LDW, LA ~ LT, and LDW ~ LT at every elevation.

**Table 2:** Standardized major axis (SMA) regression showing relationship between traits in *Parthenium hysterophorus* across an elevational gradient

Leaf trait-trait relationship	E	Estimated intercepts (*β*)	95% CI_ intercepts	Estimated slope (*α*)	95% CI_slope	*R*^2^	*p*-value
Log_LA (mm^2^) ~ Log_LDW (mg)	700	1.81	1.65–1.98	0.76	0.68–0.85	0.77	< 0.001
	1100	1.08	0.82–1.33	1.07	0.95–1.19	0.75	< 0.001
	1500	1.55	1.43–1.66	0.87	0.81–0.94	0.915	< 0.001
	1800	1.49	1.39–1.59	0.93	0.88–0.99	0.96	< 0.001
Log_LWC (mg) ~ Log_LDW (mg)	700	−2.3	−2.51– −2.09	0.89	0.79–0.99	0.75	< 0.001
	1100	−2.79	−2.95– −2.64	1.15	1.07–1.22	0.92	< 0.001
	1500	−2.56	−2.71– −2.41	1.00	0.93–1.09	0.88	< 0.001
	1800	−2.44	−2.61– −2.26	1.02	0.93–1.12	0.87	< 0.001
Log_LA (mm^2^) ~ Log_LT (mm)	700	4.94	4.59–5.29	2.18	1.75–2.71	0.04	0.07
	1100	5.62	5.17–6.06	3.83	3.16–4.65	0.27	< 0.001
	1500	5.81	5.29–6.33	3.59	2.94–4.37	0.21	< 0.001
	1800	5.75	5.11–6.40	3.86	3.00–4.96	0.07	0.04
Log_LDW (mg) ~ Log_LT (mm)	700	4.10	3.69–4.52	2.86	2.35–3.48	0.23	< 0.001
	1100	4.25	3.90–4.59	3.59	3.06–4.22	0.49	< 0.001
	1500	4.88	4.35–5.42	4.11	3.44–4.92	0.36	< 0.001
	1800	4.58	3.89–5.27	4.15	3.23–5.32	0.08	0.03

Data is reported up to only two decimal places

*Abbreviations*: *LA* Leaf area, *LDW* Leaf dry weight, *LWC* Leaf water content, *LT* Leaf thickness, *E* Elevation

## Discussion

The current investigation revealed alterations in leaf functional characteristics of *P. hysterophorus* across an elevation range of 700 to 1,800 m. The leaf thickness, chlorophyll *a*, total chlorophyll and carotenoid content, and leaf nitrogen and phosphorus content all showed a positive correlation with increasing elevation. Nevertheless, the leaf water content (LWC), leaf dry weight (LDW), leaf mass per area (LMA), leaf dry matter content (LDMC), photosynthetic efficiency, and chlorophyll *b* exhibited a decline. The relationship between LA ~ LDW and LWC ~ LDW was more pronounced.

In mountain ecosystems, differences in leaf functional traits demonstrate the capacity of invasive species to evolve, survive, and proliferate [34]. The current investigation revealed a decline in the leaf area of *P. hysterophorus* as the elevation increased. Plants frequently use adaptive strategies to endure low temperatures, which typically include lowering heat loss and limiting the exposure of internal tissues to the cold environments. The plants at higher elevations have smaller leaf areas and thicker leaves [7]. Ke et al. [35] documented that the leaf area of 39 herbaceous species decreased with increasing altitude in the northern Qinghai-Tibetan Plateau. In our study, the SLA first declined and subsequently increased with elevation. These results contrast with those of Rixen et al. [36], who reported a general decrease in SLA with increasing elevation, and Pfennigwerth et al. [37], who found no significant relationship between SLA and elevation. SLA influences the allocation of nitrogen to photosynthetic tissues [11]. Plants with a high SLA allocate more nitrogen to photosynthesis [38]. According to Gratani [39], a high SLA and low LMA at higher elevations are associated with an elevated photosynthetic rate, shorter leaf life spans, and reduced water-use efficiency. Rising altitude and decreased leaf biomass suggest a reduced investment in photosynthetic tissue. Lower leaf dry weight (i.e., less leaf tissue) and leaf lamina area (i.e., less light interception area) indicate that *P. hysterophorus* produces smaller leaves at higher elevations. Wang et al. [40] found that plants are able to regulate temperature more effectively in high-light locations by having smaller leaves. An increasing amount of leaf thickness indicates that a species can maintain leaf tissue throughout an elevation gradient. In a mountain ecosystem, plants enhance their photosynthetic efficiency by expanding leaf thickness in response to higher levels of irradiance and water stress, as well as reducing temperature with rising elevation [41, 42]. Within the invaded areas, non-native species may occupy niches that align with or diverge from their native ranges [43]. This scenario results in the selection of traits that offer the greatest benefits to the environments they invade. Various characteristics such as growth rate, resprouting capability, leaf area, leaf N content, specific leaf area, chlorophyll content, plant height, seed mass and its size, number of reproductive branches and their distribution, biomass allocation (the ratio of above- and below-ground biomass), and alterations in microbial community have been linked to the invasiveness of non-native species [43–46]. Invasive species alter soil organic carbon (SOC) and soil nutrients [47] and are linked to increased SOC pools, especially in nutrient-deficient areas [48], which is exacerbated by climate change [49].

Photosynthetic pigments, responsible for light absorption and processing, directly impact the photosynthetic capacity of a plant. Increase in temperature, water stress, and light intensity cause chlorophyll depletion by impeding chlorophyll synthesis and accelerating the breakdown of chloroplasts [50]. The current study demonstrates that when elevation increases, the overall levels of chlorophyll and carotenoid increase, compensating for the decrease in photosynthetic efficiency. Carotenoids provide protection to chloroplasts against photodamage [51]. At higher elevations, UV-B exposure leads to the bleaching of chlorophyll and a decrease in photosynthesis [51]. Decreased temperature, lowered partial pressures of O_2_ and CO_2_, and increased diurnal temperature fluctuations all inhibit chlorophyll production [52]. The current investigation found an increase in photosynthetic pigments at intermediate altitude and a decrease at high altitude, consistent with the observations made by Wingler et al. [53] and Khan et al. [54]. Higher levels of total chlorophyll and carotenoids may offset diminishing photosynthetic efficiency of plants due to harsh environmental conditions and shorter growing seasons at higher elevations [55].

Bioclimatic and topographic factors such as precipitation seasonality, elevation, annual mean temperature, and land cover affect niche expansion [56]. Changes in climatic conditions have a significant impact on morphological and phenological traits that are highly sensitive to climate change [37]. These changes may be linked to genetic or phenotypic variations that aid in tracking climatic variations associated with elevation [57]. Leaf nutrient content in plants varies with morphological traits and growth patterns such as plant height, growth rate, leaf thickness, timing of bud formation, and senescence [37]. An elevation-dependent increase in leaf nutrients (N and P) was reported in the current study. These results corroborate those of Fisher et al. [58], who reported an increase in leaf N and P up to 1,500 m altitude in the Peruvian Andes. The correlation between leaf nutrient content and elevation can be either positive, negative, or neutral [59, 60]. In order to increase their metabolic activities, plants from colder regions typically have higher levels of N and P as an adaptive response to a cold environment [61]. The leaf N and P concentrations are temperature-sensitive and have the ability to counterbalance fluctuations in external temperature [61]. The amount of N and P in leaves is influenced by both the ability of the plant to absorb these nutrients and the bioavailability of N and P in the soil [62, 63]. Guo et al. [16] found a negative correlation between water content and leaf biomass, which supports the findings of the current study that water content decreases with elevation. The high water content of leaves is another indication of adaptation to support cell expansion for metabolic processes such as net photosynthesis and transpiration [64].

A stronger association between leaf traits along an elevational gradient demonstrates the extent to which leaf traits influence the response of plants to environmental change. The current study identified significant correlations between leaf area to leaf dry weight ratio (LA ~ LDW) and leaf water content to leaf dry weight ratio (LWC ~ LDW) along an elevational gradient. Based on the variations in the scaling relationships between dry weight and leaf area, slope values (α = 0.76–1.07) are not constant [65–67]. According to Tomlinson et al. [68], leaves are subjected to many environmental constraints over elevation gradients, including fluctuations in temperature, light intensity, nutrient availability, and water availability. Except for the elevation of 1,100 m, the leaf area in the current study exhibited a slower rate of growth compared to the leaf dry weight (mechanical tissue; α = 1). This result indicated that leaves allocate greater resources towards light-intercepting tissues as opposed to mechanical support tissues in order to optimise net carbon acquisition at higher elevations. The studies by Niklas et al. [66] and Milla and Reich [69] provide evidence in support of these claims. The variance in the relationship between leaf water content and leaf dry weight ranged from 0.89 to 1.15, except for the elevation of 700 m (α ≥ 1). In the current study, an increase in leaf dry weight was associated with a correspondingly rapid increase in leaf water content. This result contrasted with that of Niklas et al. [66], who found that several plant categories had values of α < 1. With increasing elevation, there was a slight augmentation in both leaf water content and leaf dry mass, leading to a moderate increase in leaf thickness. The rapid increase in leaf water content relative to dry weight indicates the importance of water for leaf adaptation to low temperatures and intense solar radiation at higher elevations.

## Conclusions

To summarise, our research indicates that *P. hysterophorus* has the ability to expand its range to higher altitudes by adapting its leaf functional features and resource-use strategies, thus broadening its functional niche. In mountainous regions, the upward migration and proliferation of invasive species depend on their ability to alter functional traits to adjust to the existing conditions. In order to adapt to higher elevations, *P. hysterophorus* developed smaller, denser leaves with increased SLA and leaf nutrient contents. An increase in photosynthetic pigment concentrations at higher altitudes counterbalanced the decline in photosynthetic efficiency. The findings suggest that differences in leaf traits and the correlations between different traits are important factors in maintaining the fitness and growth rate of plants under challenging conditions such as low temperatures, high irradiation, and limited resources at higher elevations. The ability of invasive plant species to adapt to new environments displays their extensive range expansion in contrasting habitats compared to their native regions. This study provides valuable insights into the phenotypic plasticity and invasive tendencies of other invasive species over different elevational gradients and in response to varying environmental conditions. Furthermore, it aids in the efficient identification and conservation of native species that are at high risk of extinction. This will facilitate speculation regarding other invasive plant species, especially those that share phylogenetic or morpho-functional similarities with *P. hysterophorus*, in addition to aiding in the prediction of its future behaviour.

### Supplementary Information

**Supplementary Material 1.**

## Data Availability

Data shall be provided by corresponding author on reasonable request.
