# Supplementary material for: Leaf functional traits and resource use strategies facilitate the spread of invasive plant Parthenium hysterophorus across an elevational gradient in western Himalayas
Source: BMC Plant Biol. 2024 Apr 2;24:234. doi: 10.1186/s12870-024-04904-0 (PMC10985864; doi:10.1186/s12870-024-04904-0)
Supplement: Supplementary file 1 — Supplementary Material 1. [file 12870_2024_4904_MOESM1_ESM.docx]

# **Supplementary material**

# **Title**

# Leaf functional traits and resource use strategies facilitate the spread of invasive plant *Parthenium hysterophorus* across an elevational gradient in western Himalayas

**Authors**

Padma Sharma^1^, Sonia Rathee^2^, Mustaqeem Ahmad^1^, Manzer H. Siddiqui^3^, Saud Alamri^3^, Shalinder Kaur^2,*^, Ravinder K. Kohli^4^, Harminder Pal Singh^1^, and Daizy R. Batish^2^,

*^1^ Department of Environment Studies, Panjab University, Chandigarh 160014, India*

*^2^ Department of Botany, Panjab University, Chandigarh 160014, India*

^3^ *Department of Botany and Microbiology, College of Science, King Saud University, Riyadh 11451, Saudi Arabia*

*^4^* *Amity University, Sector 82A, IT City, International Airport Road,****Mohali,*** *140 306, India*

**Correspondence*: Dr. Shalinder Kaur, Department of Panjab University, Chandigarh, India (E-mail: shalinder@pu.ac.in)

Table S1. The details of various parameters used, including the acronyms, calculation methods, and reference(s)

| **Traits studied** | **Acronyms with units** | **Calculation methods** | **Reference(s)** |
| --- | --- | --- | --- |
| Leaf area | (LA; mm^2^), | Through portable devices that measure leaf area by sliding over the surface of the leaf and scanning it. On the device's display, the resultant measurement is shown. (CI-202, CID Bio-Science, USA) | Hakim et al. (2023) |
| Leaf dry matter content | (LDMC; mg g^−1^), | $\frac{oven dry mass of leaf}{\begin{aligned} its water saturated \\ fresh mass \end{aligned}}$ | Pérez-Harguindeguy et al. (2016) |
| Specific leaf area | (SLA; mm^2^ mg^−1^), | $\frac{one sided area of a fresh leaf}{its oven dry mass}$ | Pérez-Harguindeguy et al. (2016) |
| Leaf mass per area | (LMA; mg mm^−2^), | $\frac{leaf dry mass}{leaf area}$ | Pérez-Harguindeguy et al. (2016) |
| Leaf thickness | (LT; mm), | Measured using a digital Vernier Calliper with an accuracy of 0.01 mm | Hakim et al. (2023) |
| Leaf dry weight | (LDW; mg). | Leaves were dried for 72 hours at 60°C in a hot air oven before being weighed with an electronic weighing balance (A&D Co., Japan; accuracy = 0.1 mg). | Hakim et al. (2023) |
| Leaf water content | (LWC; mg) | It is calculated as difference between the weight of the fresh leaf and the weight of the dry leaf.  (LWC = FW – DW) | Thakur et al. (2019) |
| Total chlorophyll content | (TChl; µg mg^−1^), | TChl = (6.45 × A_663_) + (17.72 × A_645_) | Arnon (1949) |
| Chlorophyll *a* | (Chl *a*; µg mg^−1^) | Chl *a* = (10.63 × A_663_) – (2.39 × A_645_) | Arnon (1949) |
| Chlorophyll *b* | (Chl *b*; µg mg^−1^), | Chl *b* = (20.11 × A_645_) – (5.18 × A_662_) | Arnon (1949) |
| Total carotenoid content | (TCaro; (µg mg^−1^), | TCaro = (1000 – A_470_ – 3.27 × Chl *a* – 104 × Chl *b*) | Lichtenthaler and Wellburn (1983) |

**References:**

Hakim N, Ahmad M, Rathee S, Sharma P, Kaur S, Batish DR, Singh HP (2023) Invasive *Cirsium arvense* displays different resource-use strategies along local habitat heterogeneity in the trans-Himalayan region of Ladakh. Environ Monit Assess. 195: 730. https://doi/10.1007/s10661-023-11221-w

Thakur D, Rathore N, Chawla A (2019) Increase in light interception cost and metabolic mass component of leaves are coupled for efficient resource use in the high altitude vegetation. Oikos. 128: 254-263. https://doi.org/10.1111/oik.05538

Pérez-Harguindeguy N, Diaz S, Garnier E, Lavorel S, Poorter H, Jaureguiberry P, Bret-Harte MS, Cornwell WK, Craine JM, Gurvich DE, Urcelay C, Veneklaas EJ, Reich PB, Poorter L, Wright IJ, Ray P, Enrico L, Pausas JG, de Vos AC, Buchmann N, Funes G, Quétier F, Hodgson JG, Thompson K, Morga HD, ter Steege H, Sack L, Blonder B, Poschlod P, Vaieretti MV, Conti G, Stave AC, Cornelissen JHC (2016) Corrigendum to: new handbook for standardised measurement of plant functional traits worldwide. Aust J Bot. 64: 715-716. https://doi.org/10.1071/BT12225_CO

Hiscox JD, Israelstam GF (1979) A method for the extraction of chlorophyll from leaf tissue without maceration. Can J Bot. 57: 1332-1334. https://doi.org/10.1139/b79-163.

Arnon DI (1949) Copper enzymes in isolated chloroplasts. Polyphenoloxidase in *Beta vulgaris*. Plant Physiol. 24: 1-15. https://doi.org/10.1104/pp.24.1.1.

Lichtenthaler HK, Wellburn WR (1983) Determination of total carotenoids and chlorophyll a and b of leaf extracts in different solvents. Biochem Soc Trans. 11: 591-592. https://doi/10.1042/bst0110591.
